# Encouraging long‐term survival following autophagy inhibition using neoadjuvant hydroxychloroquine and gemcitabine for high‐risk patients with resectable pancreatic carcinoma

**DOI:** 10.1002/cam4.4211

**Published:** 2021-09-24

**Authors:** Samer S. AlMasri, Mazen S. Zenati, Annissa Desilva, Ibrahim Nassour, Brian A. Boone, Aatur D. Singhi, David L. Bartlett, Lance A. Liotta, Virginia Espina, Patricia Loughran, Michael T. Lotze, Alessandro Paniccia, Herbert J. Zeh, Amer H. Zureikat, Nathan Bahary

**Affiliations:** ^1^ Department of Surgery University of Pittsburgh Pittsburgh PA USA; ^2^ Department of Surgery, Epidemiology, Clinical and Translational Science University of Pittsburgh Pittsburgh PA USA; ^3^ Department of Surgery West Virginia University Morgantown WV USA; ^4^ Department of Pathology University of Pittsburgh Pittsburgh PA USA; ^5^ Department of Surgery Allegheny Health Network Pittsburgh PA USA; ^6^ Center for Applied Proteomics and Molecular Medicine George Mason University Manassas VA USA; ^7^ Department of Immunology University of Pittsburgh Pittsburgh PA USA; ^8^ Department of Bioengineering University of Pittsburgh Pittsburgh PA USA; ^9^ Department of Surgery University of Texas Southwestern Dallas TX USA; ^10^ Department of Internal Medicine University of Pittsburgh PA USA

**Keywords:** autophagy, hydroxychloroquine, neoadjuvant, overall survival, pancreatic cancer

## Abstract

**Introduction:**

Preoperative autophagy inhibition with hydroxychloroquine (HCQ) in combination with gemcitabine in pancreatic adenocarcinoma (PDAC) has been shown to be safe and effective in inducing a serum biomarker response and increase resection rates in a previous phase I/II clinical trial. We aimed to analyze the long‐term outcomes of preoperative HCQ with gemcitabine for this cohort.

**Methods:**

A review of patients enrolled between July 2010 and February 2013 in the completed phase I/II single arm (two doses of fixed‐dose gemcitabine (1500 mg/m^2^) in combination with oral hydroxychloroquine administered for 31 consecutive days until the day of surgery for high‐risk pancreatic cancer) was undertaken. Progression‐free survival (PFS) and overall survival analysis (OS) using Kaplan–Meier estimates were performed.

**Results:**

Of 35 patients initially enrolled, 29 patients underwent surgical resection (median age at diagnosis: 62 years, 45% females). Median duration of follow‐up was 7.5 years. There was a median 15% decrease in the serum CA19‐9 levels following completion of neoadjuvant therapy and 83% of the cohort underwent a pancreaticoduodenectomy, 7 (24%) patients had a concomitant venous resection. On histopathology, 14 (48%) patients had at least a partial treatment response. The median PFS and OS were 11 months (95% Confidence interval [CI]: 7–28) and 31 months (95% CI: 13–47), respectively, while 9 (31%) patients survived beyond 5 years from diagnosis; a rate that compares very favorably with contemporaneous series.

**Conclusion:**

Compared to historical data, neoadjuvant autophagy inhibition with HCQ plus gemcitabine is associated with encouraging long‐term survival for patients with PDAC.

## Graphical abstract

Preoperative autophagy inhibition with hydroxychloroquine enhances the therapeutic efficacy of neoadjuvant chemotherapy leading to a sustained and improved survival benefit in high‐risk pancreatic cancer.

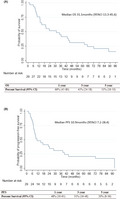

## 1 INTRODUCTION

Despite recent advances in multimodality therapy for the management of pancreatic ductal adenocarcinoma (PDAC), the prognosis for PDAC remains dismal as only 9% of patients survive beyond 5 years from diagnosis.^1^ Following curative‐intent resection and adjuvant chemotherapy, 50% of patients will experience disease recurrence within 2 years.^1^, ^2^ Adjuvant therapy has been shown to improve the overall survival (OS).^3^, ^4^, ^5^ Yet, the role of neoadjuvant therapy (NAT)––particularly in the setting of resectable PDAC––remains controversial and its utilization in the United States remains low.^6^ A recent systematic review and meta‐analysis of randomized controlled trials demonstrated that survival for patients with resectable or borderline resectable PDAC––who received NAT––was 30% higher than patients who underwent upfront surgical resection, irrespective of the NAT protocol.^7^ These findings support the utility of NAT for PDAC.

Autophagy is a cellular survival mechanism by which the cell recycles damaged organelles and structural proteins under normal circumstances and in response to adverse environmental conditions such as hypoxia, nutritional deprivation, and therapeutic stress.^8^, ^9^ Autophagy is a crucial step in pancreatic carcinogenesis as it allows cancer cells to evade apoptosis and develop chemoresistance, which ultimately leads to diminished survival.^10^, ^11^, ^12^, ^13^ Hydroxychloroquine (HCQ) belongs to a class of pharmacological drugs that block acidification of lysosomes, inhibiting a late step in autophagy.^14^, ^15^, ^16^ Due to its wide therapeutic index, high oral bioavailability, and low cost, its utility in cancer therapy is being increasingly explored.^8^, ^15^, ^16^ Two previous clinical trials have evaluated the role of HCQ in combination with gemcitabine alone or with nab‐paclitaxel in the neoadjuvant setting for potentially resectable PDAC.^15^, ^16^ These trials demonstrated HCQ to be a component of a safe and well‐tolerated treatment regimen for PDAC patients that enhances the efficacy of NAT.

In this study, we performed a secondary analysis of a single arm, prospective, phase I/II clinical trial that explored preoperative autophagy inhibition with HCQ in combination with gemcitabine for biopsy proven “high‐risk” PDAC. We hypothesized that this regimen would be associated with encouraging long‐term survival.

## 2 METHODS

### 2.1 Patient population and data collection

Patients enrolled in the previously completed clinical trial who underwent surgical resection were identified. This trial was a phase I/II prospective study evaluating preoperative gemcitabine plus HCQ.^15^ Histologic confirmation of malignancy was required through endoscopic ultrasound (EUS)‐guided biopsy before enrollment. Patients included were predicted to have limited survival following surgical resection based on a validated model that classified patients as high‐risk for residual positive margins following surgical resection.^17^ The criteria included in this model were: evidence of vascular involvement on computed tomography (CT) scan, and/or a primary tumor size ≥2.6 cm in greatest dimensions on EUS, and/or any evidence of lymph node involvement on preoperative EUS staging (AJCC 8th stage IIB or higher).^18^

Data collected included demographic, perioperative outcomes, and clinico‐pathological variables. The overall performance status of the study cohort was determined by the Eastern Cooperative Oncology Group (ECOG) scale^19^ and Charlson Age Comorbidity Index (CCI).^20^ Carbohydrate antigen 19‐9 (CA19‐9) levels at the time of diagnosis and post‐completion of NAT were documented. Pathologic variables retrieved included differentiation grade, lympho‐vascular and perineural invasion, T stage, N stage, and AJCC 8th edition pathologic stage; margins ≤1 mm were reclassified as positive residual margins.^18^ Correlative autophagy makers were assessed to gauge treatment response to HCQ as outlined in the Data S1. The pre‐ and post‐treatment serum ELISA levels of HMGB1 and peripheral blood mononuclear cell (PBMC) LC3‐II immunofluorescent staining were evaluated. Moreover, the resected pancreatic tumor specimens were immunohistochemically stained for several autophagic markers including Beclin 1, ATG7, and CD68. Patients who had ≥51% increase in their LC3‐II staining were classified as having a positive response to HCQ congruent to what was reported in the original clinical trial. A similar cut‐off was chosen to stratify response to HCQ either as a percentage serum increase following completion of NAT for HMGB1 or percentage of cancer cells staining for the autophagic markers Beclin 1, ATG7, and CD68 in the resected tumor specimen.

Pathologic tumor response to NAT was categorized into either none/poor, mild‐moderate, or complete/near‐complete response. The “none” group corresponded to an Evans grade of I or a College of American pathologists (CAP) grade of III; the mild‐moderate response group correspond to an Evans grade of IIA or IIB and a CAP score of II and lastly, the complete/near‐complete response group corresponded to an Evans grade of III or IV and a CAP score of 1 or 0.^21^, ^22^ The diagnosis of recurrence was established based on cross‐sectional imaging and classified as either local‐first, distant‐first, or local *and* distant‐first recurrence. The regimen and duration of adjuvant and salvage chemotherapy were also recorded. Lastly, the date of death or last follow‐up was documented.

The primary endpoint for this study was to evaluate the OS and progression‐free survival (PFS) measured from the date of diagnosis. Secondary endpoints included response to NAT as assessed by the CA19‐9 serum level change following preoperative therapy, histopathologic response, and correlative studies of autophagy. The CA19‐9 level change was used as a clinical surrogate marker of response.

### 2.2 Statistical analysis

Statistical analyses were performed using STATA 16 (StataCorp LP). We used descriptive statistics to summarize baseline patient characteristics and clinico‐pathological variables; continuous variables are reported using median and interquartile range (IQR) while categorical variables are reported using relative frequencies with corresponding (percentages). Survival was characterized using Kaplan–Meier estimates, and log‐rank tests were performed to compare the survival functions. Patients with at least a partial histopathologic treatment response were grouped and their survival functions were compared to those who lacked a pathologic response. All tests were two‐sided and a *p* value <0.05 was considered statistically significant.

## 3 RESULTS

### 3.1 Patient demographics, histopathology, and outcomes

**FIGURE 1:**
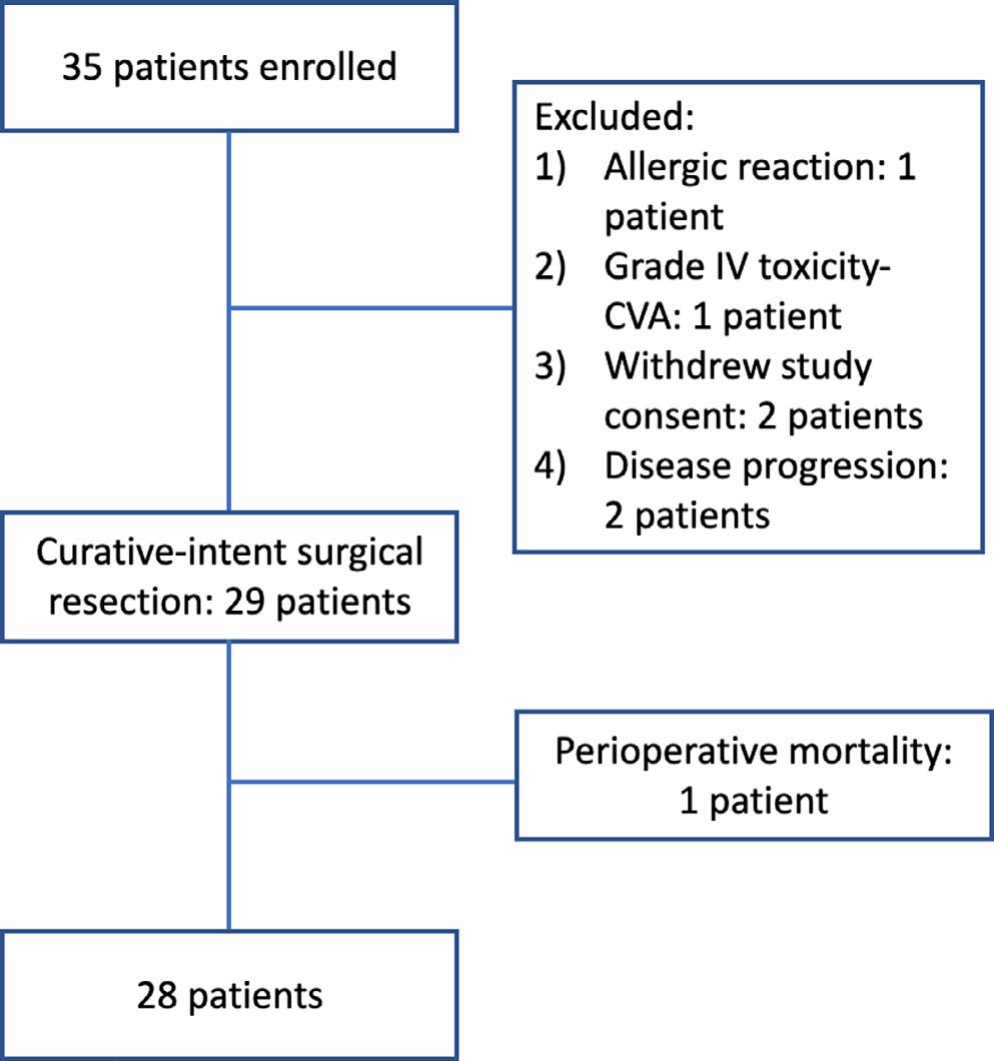
Patient enrollment

Between July 2010 and February 2013, 35 patients were enrolled in the clinical protocol. As shown in Figure 1, 29 of 35 patients (83%) ultimately underwent surgical resection. In total, six patients were excluded: two patients withdrew study consent prior to initiation of therapy, one patient developed a grade 4 toxicity (cerebrovascular accident) deemed to be unrelated to the treatment regimen, one patient developed an allergic rash after the first dose of HCQ, and two patients were found to have unresectable disease secondary to liver metastasis at the time of surgical exploration. Those six patients were excluded from this analysis.

The median age at diagnosis was 62 years, 45% were females, and the majority (57%) had an ECOG performance status of 1 (Table 1). The median tumor size on preoperative EUS staging was 3.1 cm. On pre‐treatment CT, 17 (59%) patients had a resectable disease, 12 (41%) patients had a borderline‐resectable disease due to venous invasion in 11 (92%), and celiac axis abutment in 1 (8%) patient. Twenty‐four patients (83%) underwent a Whipple procedure (seven requiring venous resection) and five (17%) patients underwent a distal pancreatectomy with splenectomy, of which one patient required celiac axis resection (modified Appleby‐type operation). On final histopathology, 16 (55%) patients had a T2 lesion and 14 (48%) patients had a N2 disease. Since this study utilized the AJCC 8th staging system, 48% of the cohort had stage III disease and 14 (48%) patients had positive residual margins. Of the latter group, six (43%) patients had a positive retroperitoneal margin, five (36%) patients had a positive vascular groove margin, and three (21%) patients had a positive pancreatic neck margin.

**TABLE 1:** Patient demographics, clinico‐pathological variables

Characteristic	Value
Age at diagnosis, years	64 ± 10
Gender (female)	13 (45)
BMI (kg/m^2^)	28.9 (23.4–31.9)
ECOG	
0	12 (43)
1	17 (57)
Radiographic stage at diagnosis	
Resectable	17 (59)
Borderline‐resectable	12 (41)
CA19‐9 pre‐NAT, U/ml	375 (60–758)
CA19‐9 post‐NAT, U/ml	131 (31–440)
CA19‐9% change	−15% (−48% to 18%)
Operation performed	
Pancreaticoduodenectomy	24 (83)
Distal pancreatectomy	4 (14)
DP‐CAR	1 (3)
Tumor size, cm	3.5 (2.7–4.1)
T stage	
1	5 (17)
2	16 (55)
3	8 (28)
N stage	
0	8 (28)
1	7 (24)
2	14 (48)
pAJCC stage	
I	6 (21)
II	9 (31)
III	14 (48)
Grade	
Moderately differentiated	17 (69)
Poorly differentiated	12 (41)
Lympho‐vascular invasion	22 (76)
Perineural invasion	24 (83)
Pathologic response	
None	15 (52)
Mild‐moderate	13 (45)
Near‐complete	1 (4)
Positive margins	14 (48)

Data are reported as mean±standard deviation, median (interquartile range), or as raw number (percentage). BMI, body mass index, ECOG, Eastern Cooperative Oncology Group classification, NAT, neoadjuvant treatment, DP‐CAR, Distal pancreatectomy‐celiac axis resection, pAJCC, Pathologic American Joint Committee on Cancer Stage.

The median length of postoperative hospital stay was 8 (7–10) days (Table 2). Major complications (classified as a Clavien‐Dindo score ≥ 2) were observed in four (14%) patients. There was one perioperative mortality secondary to superior mesenteric vein thrombosis following venous resection and reconstruction, resulting in ischemic bowel and multisystem organ failure.

**TABLE 2:** Clinical outcomes and postoperative therapy

Characteristic	Value
Postoperative outcomes	
Length of stay, days	8 (7–10)
Clavien‐Dindo score >2	4 (14)
90‐day mortality	1 (3%)
Adjuvant therapy	
Adjuvant chemotherapy receipt	24 (83%)
Adjuvant chemotherapy number of cycles	5.5 (4–6)
Adjuvant radiation	8 (28%)
Recurrence data	
Diagnosis of recurrence	22 (79%)
Local first recurrence	10 (46%)
Distant first recurrence	8 (36%)
Local and distance recurrence	4 (18%)
Salvage therapy	
Salvage therapy receipt	17 (61%)
Salvage therapy regimen	
Gemcitabine based	6 (38)
5‐FU based	6 (38)
Crossover	4 (4)
Salvage chemotherapy number of cycles	3.5 (2.0–11.5)
Palliative radiation therapy	5 (18%)

Data are reported as median (interquartile range) or as raw number (percentage).

Adjuvant chemotherapy was administered to 24 (83%) patients. The most frequently used regimen was gemcitabine monotherapy in 20 (83%) patients, while 8 (28%) patients received adjuvant radiation (2 of those received stereotactic body radiotherapy as the only adjuvant therapy). The median length of follow‐up for the overall study population was 7.5 years during which 22 (79%) patients developed disease recurrence that was detected as a local‐first recurrence in 46%, a distant‐first recurrence in 36%, and concomitant local and distant‐recurrence in 18%. In total, 61% of patients received salvage therapy, 38% of patients were gemcitabine based, and 38% of patients where 5‐fluorouracil based.

### 3.2 Tumor marker and histopathologic response

**FIGURE 2:**
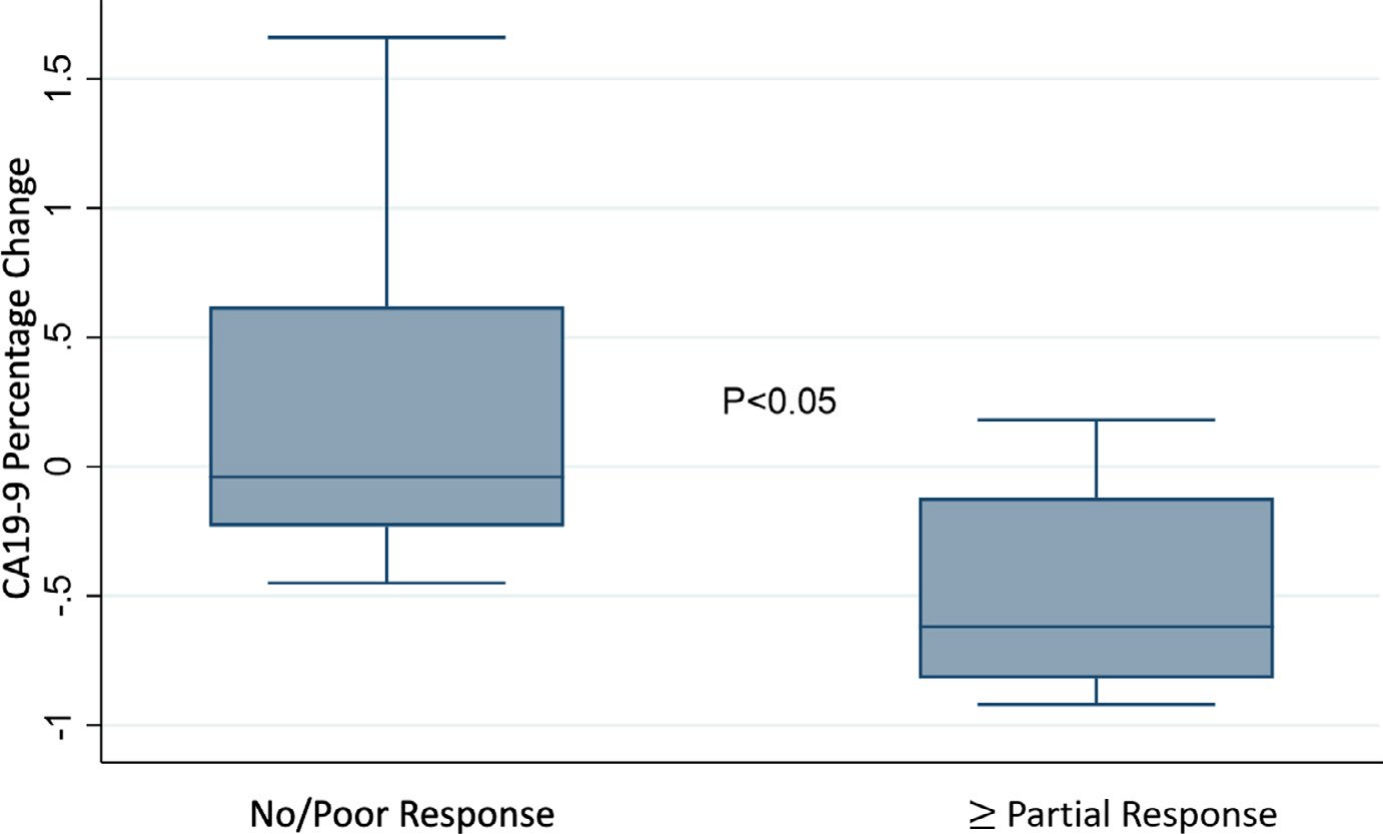
CA19‐9 percentage change in response to neoadjuvant therapy stratified by histopathologic treatment response

The median CA19‐9 level at diagnosis was 375 (60–758) IU/ml, while the median level following completion of NAT was 131 (31–440) IU/ml. This translated to a median 15% (−48%, 18%) decrease in the CA19‐9 level in response to treatment. On histopathologic assessment (Table 1), 14 (48%) patients had at least a partial histopathologic treatment response; 13 (93%) patients had a mild‐moderate treatment response, and 1 (7%) patient had a near‐complete response. Fifteen patients (52%) had a poor/no treatment response. For the latter group, the median change in CA19‐9 level was −4% (−23%, 62%), while for the former group, the median decrease in CA19‐9 level was −62% (−82%, −12%) (*p*<0.05) (Figure 2).

Patients with no/poor treatment response had more proximal tumors (pancreatic head) (87% vs. 71%, *p *= 0.032), higher median number of positive lymph nodes (8 vs. 1, *p *= 0.011), and higher incidence of lympho‐vascular and perineural invasion compared to those with at least a partial treatment response (93% vs. 57% *p* = 0.035, 100% vs. 71% *p* = 0.042, respectively).

### 3.3 Survival analysis

**FIGURE 3:**
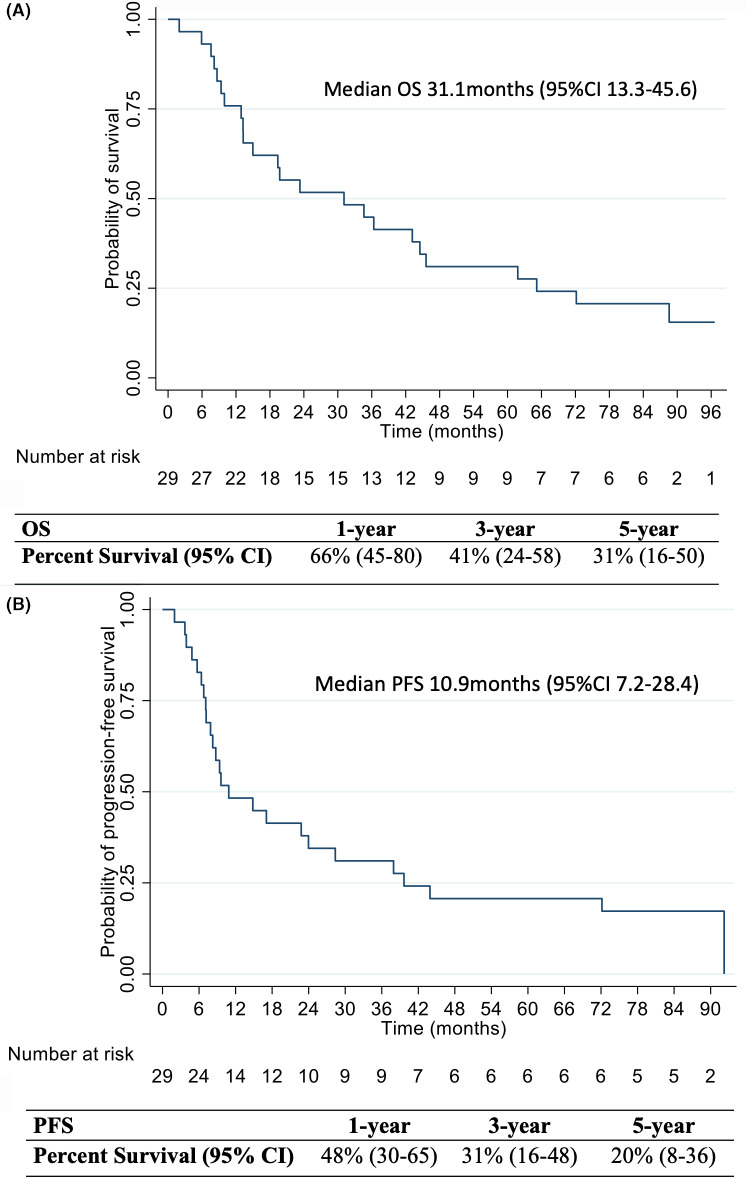
Overall survival (A) and progression‐free survival (B) with the corresponding 1‐, 3‐, and 5‐year percent OS and PFS rates

Median OS for the resected cohort was 31.1 months (95% confidence interval [CI] 13.4–45.6), and median PFS was 10.9 months (95% CI 7.2–28.4; Figure 3). The estimated 5‐year OS rate was 31% (16–47) and the 5‐year PFS rate was 20% (8–36). On univariate analysis, OS was significantly improved in patients with resectable disease at diagnosis (44.5 vs. 19.4 months for borderline disease, *p* < 0.019), ≥partial treatment response (61.8 vs. 19.4 months for none/poor histopathologic response, *p* = 0.003), and receipt of ≥ 6 cycles of adjuvant chemotherapy (61.8 vs. 13.3 months for < 6 cycles, *p* = 0.009). The median PFS was also significantly higher for patients who had at least a partial histologic response as compared to patients who lacked a treatment response (26.7 vs. 6.3 months, *p* = 0.001).

### 3.4 Correlative studies

The percent cancer cell staining for Beclin 1, ATG7, and CD68 as well as the percentage change in response to HCQ for HMGB1 and LC3‐II are reported in Table S1. There was no significant association between the percent cancer cell staining for Beclin 1, ATG7, and CD68 or the percentage change for HMGB1 and LC3‐II with the CA19‐9 level change, histopathologic tumor response, and other pathologic tumor characteristics. However, patients who had a positive response––as assessed by LC3‐II PBMCs staining of ≥51%––had an improved PFS (13 vs. 4 months, *p* = 0.046) and a trend toward an improved OS (23 vs. 10 month, *p* = 0.055) compared to patients who had <51% change. On the other hand, and although no significant association with either PFS or OS was identified for the remaining autophagic markers, patients who had ≥ 51% cancer cell staining for Beclin 1 had worsened PFS and OS rates as opposed to patients who had <51% staining (6 vs. 27 months *p* = 0.001 and 10 vs. 45 months *p* = 0.002, respectively).

## 4 DISCUSSION

This study evaluated the long‐term outcomes of a previously published clinical trial^15^ and demonstrates that preoperative treatment with HCQ in combination with gemcitabine for high‐risk PDAC is associated with encouraging long‐term overall and disease‐free survival. The median OS of the entire surgically resected cohort––from the time of diagnosis––was 31 months and nearly a third of patients were alive 5 years from diagnosis. This rate is significantly higher than what would be expected with gemcitabine monotherapy following curative‐intent surgical resection, especially for this group of high‐risk patients. In the ESPAC‐3(v2) (European Study group for Pancreatic Cancer) RCT, the median and 5‐year OS following surgical resection and gemcitabine monotherapy were 25.5 months (95%CI 22.7–27.9) and 17.5% (95% CI 14.0–21.2), respectively. While in the ESPAC‐4 RCT, they were 23.6 months (95%CI 21.4–26.4) and 16.3% (95%CI 10.2–23.7), respectively.^4^, ^23^ In the CONKO‐001 (Charité Onkologie) RCT, the median and 5‐year OS following R0 surgical resection and adjuvant gemcitabine were 22.8 months (95% CI 18.5–27.2 months) and 20.7% (95% CI 14.7%–26.6%) while in the CONKO‐005 RCT, they were 26.5 months (95%CI 22.4–30.6) and 20%, respectively.^3^, ^24^ These findings further support the role of autophagy inhibition with HCQ in combination with neoadjuvant chemotherapy for potentially resectable PDAC.

Notably, nearly half of this cohort (48%) experienced at least a partial histopathologic response. This subset was found to have a median OS of nearly 5 years; an outcome rarely associated with pancreatic cancer. Our findings suggest that histopathologic response has major prognostic implications, and this corroborates with the findings by White et al,^25^ where the authors found that patients who had a large residual tumor load (Evans I/IIa) had worse survival when compared to those who had a moderate (Evans IIb) or complete histopathologic response (Evans III/IV). This is in contrast with the findings presented by Chatterjee and colleagues^26^; where although the authors concluded that the grade and extent of residual tumor have crucial prognostic implications in PDAC patients following NAT, they found no significant difference in the median OS among patients with poor/no response (CAP III, Evans I/IIA) versus those who had a moderate response (CAP II, Evans IIb). Therefore, the combination of HCQ with gemcitabine in the neoadjuvant setting seems to induce at least a moderate histopathologic response in a significant proportion of patients and this appeared to translate into a sustained and improved survival.

In an open label, phase II, randomized clinical trial, Karasic et al^27^ evaluated the role of HCQ, in addition to gemcitabine and nab‐paclitaxel, in the setting of metastatic or advanced PDAC. Although there was a significant increase in the overall response rates among the HCQ arm (21% vs. 38%, *p* = 0.047), this did not translate to an improvement in the primary endpoint of 1‐year OS. This finding can be partly explained by the advanced disease stage of this cohort. The authors did conclude, however, that the improvement in response rates seen with autophagy inhibition may be most useful in the neoadjuvant setting, where tumor response might enable surgical resection. Further trials may be needed to evaluate autophagy inhibition as a novel treatment strategy for recurrent/metastatic PDAC.

In a recently published phase II clinical trial, patients with potentially resectable PDAC were randomized to gemcitabine and nab‐paclitaxel alone or with HCQ followed by surgical resection. This trial confirmed the previous clinical observation of the original trial that the addition of HCQ to neoadjuvant gemcitabine/*nab*‐paclitaxel is safe and leads to a significant increase in the proportion of patients experiencing an Evans grade IIb or higher histopathologic response (*p* < 0.001).^16^ Although this did not translate to improvement in the OS and PFS rates, the trial was not powered to detect survival differences and adjuvant treatment was not controlled for. In our study population, 83% of the cohort received adjuvant chemotherapy, the majority of which were gemcitabine‐based, as the study was completed prior to FOLFIRNOX becoming the standard of care in the adjuvant setting.^5^

To delineate the potential benefit of HCQ in the neoadjuvant setting, this study correlated with the observed clinical outcomes with several markers of autophagy that are known to increase in response to HCQ.^28^, ^29^, ^30^, ^31^ A ≥51% increase in LC3‐II level––assessed by immunofluorescent staining of PBMCs pre‐ and post‐treatment––correlated with an improved OS and PFS. This suggests that autophagy inhibition played a critical role in the observed clinical outcomes of NAT with HCQ for pancreatic cancer patients. However, no significant correlation was observed with HMGB1, ATG7, and CD68 histologic measures. Interestingly, an increase in Beclin 1 expression––as reflected by the percent cancer cells staining in the resected pancreatic specimen––correlated with a worsened PFS and OS. Elevated Beclin 1 expression has been reported to be a favorable prognostic biomarker in several other solid tumors.^32^, ^33^, ^34^ Conversely, limited evidence suggests an opposite correlation between Beclin 1 expression and clinical outcomes similar to the findings presented in this study.^35^, ^36^ The underlying mechanism for the differing prognostic impact of Beclin 1 expression may depend on the intrinsic properties of the tumor subtype and the treatment regimen. Nevertheless, further evidence is needed to assess the prognostic impact of Beclin 1 expression levels in pancreatic cancer.

This analysis further contributes to the growing body of evidence surrounding the role of autophagy inhibition in pancreatic cancer as an important therapeutic target, especially in the neoadjuvant setting.^36^, ^37^ A recent exciting study^38^ and associated commentaries^39^, ^40^, ^41^, ^42^, ^43^, ^44^, ^45^ have noted that enhanced autophagy in the setting of pancreatic cancer enables degradation of Class I major histocompatibility (MHC) molecules, critical for recognition by CD8+ T cells. Indeed, a previously published randomized study suggests that autophagy inhibition with HCQ promotes a much higher T‐cell infiltrate, consistent with a significant immune effect.^16^ Although autophagy may also enhance the bioenergetics necessary to enable completion of the apoptotic process or the ferroptotic cell death,^46^ the preponderance of available evidence––including the promising late survival in our patient population––suggests that its continued evaluation, particularly in the neoadjuvant setting is warranted. Lastly, it appears clear that autophagy also limits immune effector function by degrading proapoptotic molecules and/or limiting TNF signaling.^47^, ^48^

Our study has several limitations that are primarily driven by its retrospective study design and small sample size. Nevertheless, we sought to evaluate the long‐term outcomes of a single arm clinical trial that assessed a novel treatment strategy in high‐risk PDAC patients and found that the addition of HCQ to gemcitabine resulted in a median OS of 31 months with nearly a third of the cohort surviving beyond 5 years from diagnosis; a rate much higher than would be expected for this patient population.

## 5 CONCLUSION

This study demonstrates that preoperative autophagy inhibition with HCQ is a safe and well‐tolerated treatment regimen that enhances the therapeutic efficacy of NAT and improves pathologic response rates in PDAC. This translated to a sustained and improved survival benefit compared to previously published trials in the gemcitabine monotherapy era. Planned future studies include evaluation of the role of FOLFIRINOX and HCQ compared to gemcitabine/nab‐paclitaxel/HCQ (the “PGH” regimen) in the neoadjuvant setting. The results of these studies may further facilitate the identification of a clear survival benefit for the addition of HCQ to preoperative therapy in operable pancreatic cancer.

## CONFLICT OF INTEREST

None, financial or otherwise.

## ETHICAL STATEMENT

Institution Review Board approval at the University of Pittsburgh (IRB STUDY#20040099).

## Supporting information

Table S1

Data S1

## Data Availability

The data that support the findings of this study are available upon request from the corresponding author. The data are not publicly available due to privacy or ethical restrictions.

## References

[1] Siegel RL , Miller KD , Jemal A . Cancer statistics, 2020. CA Cancer J Clin. 2020;70:7‐30. 31912902 10.3322/caac.21590

[2] McGuigan A , Kelly P , Turkington RC , et al. Pancreatic cancer: a review of clinical diagnosis, epidemiology, treatment and outcomes. W J Gastroenterol. 2018;24:4846‐4861. 10.3748/wjg.v24.i43.4846 PMC6250924 30487695

[3] Oettle H , Neuhaus P , Hochhaus A , et al. Adjuvant chemotherapy with gemcitabine and long‐term outcomes among patients with resected pancreatic cancer. JAMA. 2013;310:1473‐1481. 24104372 10.1001/jama.2013.279201

[4] Neoptolemos JP , Palmer DH , Ghaneh P , et al. European Study Group for Pancreatic Cancer . Comparison of adjuvant gemcitabine and capecitabine with gemcitabine monotherapy in patients with resected pancreatic cancer (ESPAC‐4): a multicenter, open‐label, randomized, phase 3 trial. Lancet. 2017;389:1011‐1024. 28129987 10.1016/S0140-6736(16)32409-6

[5] Conroy T , Hammel P , Hebbar M , et al. Canadian Cancer Trials Group and the Unicancer‐GI–PRODIGE Group . FOLFIRINOX or gemcitabine as adjuvant therapy for pancreatic cancer. N Engl J Med. 2018;379:2395‐2406. 30575490 10.1056/NEJMoa1809775

[6] Hashmi A , Kozick Z , Fluck M , et al. Neoadjuvant versus adjuvant chemotherapy for resectable pancreatic adenocarcinoma: a national cancer database analysis. Am Surg. 2018;84:1439‐1445. 30268172

[7] Cloyd JM , Heh V , Pawlik TM , et al. Neoadjuvant therapy for resectable and borderline resectable pancreatic cancer: a meta‐analysis of randomized controlled trials. J Clin Med. 2020;9:1129. 10.3390/jcm9041129 PMC7231310 32326559

[8] Amaravadi RK , Lippincott‐Schwartz J , Yin XM , et al. Principles and current strategies for targeting autophagy for cancer treatment. Clin Cancer Res. 2011;17:654‐666. 21325294 10.1158/1078-0432.CCR-10-2634 PMC3075808

[9] Buchser WJ , Laskow TC , Pavlik PJ , et al. Cell mediated autophagy promotes cancer cell survival. Cancer Res. 2012;72:2970‐2979. 22505650 10.1158/0008-5472.CAN-11-3396 PMC3505669

[10] Kang R , Tang D . Autophagy in pancreatic cancer pathogenesis and treatment. Am J Cancer Res. 2012;2:383‐396. 22860230 PMC3410583

[11] Yang S , Wang X , Contino G , et al. Pancreatic cancers require autophagy for tumor growth. Genes Dev. 2011;25:717‐729. 21406549 10.1101/gad.2016111 PMC3070934

[12] Mirzoeva OK , Hann B , Hom YK , et al. Autophagy suppression promotes apoptotic cell death in response to inhibition of the PI3K‐mTOR pathway in pancreatic adenocarcinoma. J Mol Med (Berl). 2011;89:877‐889. 21678117 10.1007/s00109-011-0774-y

[13] Boone BA , Zeh HJ 3rd , Bahary N . Autophagy inhibition in pancreatic adenocarcinoma. Clin Colorectal Cancer. 2018;17:25‐31. 29223362 10.1016/j.clcc.2017.10.013

[14] Livesey KM , Tang D , Zeh HJ , et al. Autophagy inhibition in combination cancer treatment. Curr Opin Investig Drugs. 2009;10:1269‐1279. 19943199

[15] Boone BA , Bahary N , Zureikat AH , et al. Safety and biologic response of pre‐operative autophagy inhibition in combination with gemcitabine in patients with pancreatic adenocarcinoma. Ann Surg Oncol. 2015;22:4402‐4410. 25905586 10.1245/s10434-015-4566-4 PMC4663459

[16] Zeh HJ , Bahary N , Boone BA , et al. A randomized phase II preoperative study of autophagy inhibition with high‐dose hydroxychloroquine and gemcitabine/nab‐paclitaxel in pancreatic cancer patients. Clin Cancer Res. 2020;26:3126‐3134. 32156749 10.1158/1078-0432.CCR-19-4042 PMC8086597

[17] Bao P , Potter D , Eisenberg DP , et al. Validation of a prediction rule to maximize curative (R0) resection of early‐stage pancreatic adenocarcinoma. HPB (Oxford). 2009;11:606‐611. 20495714 10.1111/j.1477-2574.2009.00110.x PMC2785957

[18] Kakar S , Pawlik TM , Allen PJ , et al. Pancreas (Endocrine and Exocrine). In: Amin MB , ed. AJCC Cancer Staging Manual. Springer Nature; 2017.

[19] Oken MM , Creech RH , Tormey DC , et al. Toxicity and response criteria of the eastern cooperative oncology group. Am J Clin Oncol. 1982;5:649‐656. 7165009

[20] Charlson M , Szatrowski TP , Peterson J , et al. Validation of a combined comorbidity index. J Clin Epidemiol. 1994;47:1245‐1251. 7722560 10.1016/0895-4356(94)90129-5

[21] Evans DB , Rich TA , Byrd DR , et al. Preoperative chemoradiation and pancreaticoduodenectomy for adenocarcinoma of the pancreas. Arch Surg. 1992;127:1335‐1339. 1359851 10.1001/archsurg.1992.01420110083017

[22] Ryan R , Gibbons D , Hyland JMP , et al. Pathological response following long‐course neoadjuvant chemoradiotherapy for locally advanced rectal cancer. Histopathology. 2005;47:141‐146. 16045774 10.1111/j.1365-2559.2005.02176.x

[23] Neoptolemos J , Büchler M , Stocken DD , et al. ESPAC‐3(v2): a multicenter, international, open‐label, randomized, controlled phase III trial of adjuvant 5‐fluorouracil/folinic acid (5‐FU/FA) versus gemcitabine (GEM) in patients with resected pancreatic ductal adenocarcinoma. J Clin Oncol. 2009;27:LBA4505.

[24] Sinn M , Bahra M , Liersch T , et al. CONKO‐005: adjuvant chemotherapy with gemcitabine plus erlotinib versus gemcitabine alone in patients after R0 resection of pancreatic cancer: a multicenter randomized phase III trial. J Cin Oncol. 2017;35:3330‐3337. 10.1200/JCO.2017.72.6463 28817370

[25] White RR , Xie HB , Gottfried MR , et al. Significance for histological response to preoperative chemoradiotherapy for pancreatic cancer. Ann Surg Oncol. 2005;12:214‐221. 15827813 10.1245/ASO.2005.03.105

[26] Chatterjee D , Katz MH , Rashid A , et al. Histologic grading of the extent of residual carcinoma following neoadjuvant chemoradiation in pancreatic ductal adenocarcinoma. Cancer. 2011;118:3182‐3190. 22028089 10.1002/cncr.26651 PMC3269538

[27] Karasic TB , O’Hara MH , Loaiza‐Bonilla A , et al. Effect of gemcitabine and nab‐paclitaxel with or without hydroxychloroquine on patients with advanced pancreatic cancer: a phase 2 randomized clinical trial. JAMA Oncol. 2019;5:993‐998. 31120501 10.1001/jamaoncol.2019.0684 PMC6547080

[28] Klionsky DJ , Abdalla FC , Abeliovich H , et al. Guidelines for the use and interpretation of assays for monitoring autophagy. Autophagy. 2012;8:445‐544. 22966490 10.4161/auto.19496 PMC3404883

[29] Wolpin BM , Rubinson DA , Wang X , et al. Phase II and pharmacodynamic study of autophagy inhibition using hydroxychloroquine in patients with metastatic pancreatic adenocarcinoma. Oncologist. 2014;19:637‐638. 24821822 10.1634/theoncologist.2014-0086 PMC4041680

[30] Ramser B , Kokot A , Metze D , et al. Hydroxychloroquine modulates metabolic activity and proliferation and induces autophagic cell death of human dermal fibroblasts. J Invest Dermatol. 2009;129:2419‐2426. 19357706 10.1038/jid.2009.80

[31] Xu T , Jiang L , Wang Z . The progression of HMGB1‐induced autophagy in cancer biology. Onco Targets Ther. 2018;12:365‐377. 30643434 10.2147/OTT.S185876 PMC6317470

[32] Li BX , Li CY , Peng RQ , et al. The expression of beclin 1 is associated with favorable prognosis in stage IIIB colon cancers. Autophagy. 2009;5:303‐306. 19066461 10.4161/auto.5.3.7491

[33] Pirtoli L , Cevenini G , Tini P , et al. The prognostic role of Beclin 1 protein expression in high‐grade gliomas. Autophagy. 2009;5:930‐936. 19556884 10.4161/auto.5.7.9227

[34] Shi Y‐H , Ding Z‐B , Zhou J , et al. Prognostic significance of Beclin 1‐dependent apoptotic activity in hepatocellular carcinoma. Autophagy. 2009;5:380‐382. 19145109 10.4161/auto.5.3.7658

[35] Giatromanolaki A , Koukourakis MI , Koutsopoulos A , et al. High Beclin 1 expression defines a poor prognosis in endometrial adenocarcinomas. Gynecol Oncol. 2011;123:147‐151. 21741077 10.1016/j.ygyno.2011.06.023

[36] Wan X‐B , Fan X‐J , Chen M‐Y , et al. Elevated Beclin 1 expression is correlated with HIF‐1a in predicting poor prognosis of nasopharyngeal carcinoma. Autophagy. 2010;6:395‐404. 20150769 10.4161/auto.6.3.11303

[37] Li J , Chen X , Kang R , et al. Regulation and function of autophagy in pancreatic cancer. Autophagy. 2020;20:1‐22. 10.1080/15548627.2020.1847462 PMC8632104 33161807

[38] Piffoux M , Eriau E , Cassier PA . Autophagy as a therapeutic target in pancreatic cancer. Br J Cancer. 2021;124:333‐344. 32929194 10.1038/s41416-020-01039-5 PMC7852577

[39] Yamamoto K , Venida A , Yano J , et al. Autophagy promotes immune evasion of pancreatic cancer by degrading MHC‐I. Nature. 2020;581:100‐105. 32376951 10.1038/s41586-020-2229-5 PMC7296553

[40] Bozic M , Wilkinson S . Selective autophagy conceals the enemy: why cytotoxic T cells don't (MH)C pancreatic cancer. Mol Cell. 2020;79:6‐8. 32619471 10.1016/j.molcel.2020.06.009

[41] Huang X , Zhang X , Bai X , et al. Eating self for not be eaten: pancreatic cancer suppresses self‐immunogenicity by autophagy‐mediated MHC‐I degradation. Signal Transduct Target Ther. 2020;5:94. 32532955 10.1038/s41392-020-0209-8 PMC7293222

[42] Yamamoto K , Venida A , Perera RM , et al. Selective autophagy of MHC‐I promotes immune evasion of pancreatic cancer. Autophagy. 2020;16:1524‐1525. 32459143 10.1080/15548627.2020.1769973 PMC7469632

[43] Kroemer G , Zitvogel L . Seeking cellular fitness and immune evasion: autophagy in pancreatic carcinoma. Cancer Cell. 2020;37:759‐760. 32470391 10.1016/j.ccell.2020.05.009

[44] Hindson J . PDAC resistance to immunotherapy—a role for autophagy? Nat Rev Gastroenterol Hepatol. 2020;17:382. 10.1038/s41575-020-0318-4 32433530

[45] Maitra A . Pancreatic cancer hidden in plain sight. Nature. 2020;581:34‐35. 32322029 10.1038/d41586-020-01103-3

[46] Li C , Zhang Y , Liu J , et al. Mitochondrial DNA stress triggers autophagy‐dependent ferroptotic death. Autophagy. 2020;18:1‐13. 10.1080/15548627.2020.1739447 PMC8078708 32186434

[47] Baginska J , Viry E , Berchem G , et al. Granzyme B degradation by autophagy decreases tumor cell susceptibility to natural killer‐mediated lysis under hypoxia. Proc Natl Acad Sci USA. 2013;110:17450‐17455. 24101526 10.1073/pnas.1304790110 PMC3808626

[48] Young TM , Reyes C , Pasnikowski E , et al. Autophagy protects tumors from T cell‐mediated cytotoxicity via inhibition of TNFα‐induced apoptosis. Sci Immunol. 2020;5:eabb9561. 33443027 10.1126/sciimmunol.abb9561

