# Supplementary material for: Encouraging long‐term survival following autophagy inhibition using neoadjuvant hydroxychloroquine and gemcitabine for high‐risk patients with resectable pancreatic carcinoma
Source: Cancer Med. 2021 Sep 24;10(20):7233–41. doi: 10.1002/cam4.4211 (PMC8525088; doi:10.1002/cam4.4211)
Supplement: Supplementary file 1 — Table S1 [file CAM4-10-7233-s002.docx]

|  | **Correlative Studies - Serum** | | | **Correlative Studies - PBMCs** | | | **Correlative Studies-Resected Pancreas** | | | | |
| --- | --- | --- | --- | --- | --- | --- | --- | --- | --- | --- | --- |
|  | **HMGB1 ELISA ng/mL** | | | **LC3-II Cytospin Staining** | | | **Beclin 1** | | **ATG 7** | | **CD68** |
| **Pt ID** | **Pre Tx** | **Post Tx** | **% Change** | **Pre Tx** | **Post Tx** | **% Change** | **Staining intensity (0-4)** | **% Cancer cell staining** | **Staining intensity (0-4)** | **% Cancer cell staining** | **% Cancer cell staining** |
| **2** |  |  |  | 0.16 | 100.11 | 62469% | 2+ | 73.1 | 1+ | **58.3** | 32.26 |
| **3** |  |  |  | 52.89 | 565.37 | 969% | 2+ | 61.4 | 2+ | **60.7** | 50.00 |
| **4** |  |  |  | 339.65 | 410.13 | 21% | 0 - 1+ | 11.9 | <1+ | **21.2** | 63.49 |
| **5** |  |  |  | 261.08 | 395.45 | 51% |  |  |  |  |  |
| **7** | 2.91 | 4.96 | 70% | 921.51 | 3725.61 | 304% |  |  |  |  |  |
| **8** | 2.71 | 0.10 | -96% | 1001.04 | 278.65 | -72% | 0 - 1+ | 52.9 | 1+ | **52.2** | 19.23 |
| **9** | 6.01 | 13.25 | 120% | 1 | 353.29 | 35229% | 1+ | 25.0 | 1+ | **28.6** | 61.76 |
| **10** | 17.14 | 11.80 | -31% | 1681.6 |  |  | 2+ - 3+ | 82.5 | 2+ | **83.3** | 88.89 |
| **11** | 32.86 | 47.88 | 46% | 959.05 | 510.08 | -47% | 1+ - 2+ | 29.7 | 1+ | **75.0** | 75.76 |
| **12** | 6.70 | 4.74 | -29% | 169.83 | 472.88 | 178% | 2+ | 60.8 | 1+ | **48.3** | 43.33 |
| **13** | 10.75 | 0.57 | -95% |  |  |  | 1+ | 40.9 | 1+ - 2+ | **109.1** | 36.67 |
| **14** | 17.03 | 12.50 | -27% |  |  |  | 1+ | 49.2 | <1+ | **14.8** | 21.05 |
| **15** | 7.64 | 20.44 | 168% | 449.88 | 1437.86 | 220% | 0 - 1+ | 8.3 | 1+ - 2+ | **78.1** | 69.23 |
| **16** | 19.11 | 4.20 | -78% | 13.37 | 576.24 | 4210% |  |  |  |  |  |
| **17** |  |  |  | 542.8 |  |  | 2+ | 71.4 | 2+ | **83.3** | 33.33 |
| **18** | 5.05 | 6.03 | 19% | 131.02 | 91.38 | -30% | 1+ | 42.4 | 2+ | **66.7** | 96.77 |
| **19** |  |  |  | 169.9 |  |  | 1+ | 19.0 | 1+ | **70.8** | 66.67 |
| **20** |  |  |  | 304.0 |  |  | 2+ - 3+ | 47.4 | 0 - 1+ | **29.6** | 30.77 |
| **21** | 10.69 | 11.24 | 5% | 1604.73 | 23.78 | -99% | 1+ - 2+ | 43.2 | 2+ | **15.8** | 80.56 |
| **22** | 5.17 | 11.58 | 124% | 140.7 |  |  | 1+ - 3+ | 23.1 | 2+ | **91.9** | 59.26 |
| **23** |  |  |  | 182.9 |  |  | 2+ - 3+ | 69.4 | 2+ | **88.9** | 50.00 |
| **24** |  |  |  | 299.6 |  |  | 2+ | 40.0 | 3+ | **88.0** | 70.37 |
| **25** |  |  |  | 29.1 |  |  | 1+ - 2+ | 39.0 | 1+ | **53.8** | 34.62 |
| **27** |  |  |  |  |  |  |  |  |  |  |  |
| **29** | 11.31 | 7.62 | -33% | 4.57 | 130.92 | 2765% | 2+ | 27.3 | 0 - 1+ | **22.2** | 87.50 |
| **30** |  |  |  |  | 57.7 |  | 1+ - 3+ | 52.9 | 2+ | **92.9** | 25.00 |
| **31** | 19.26 | 11.15 | -42% | 89.5 | 3.92 | -96% | 0-3+ | 75.0 | 1+ | **90.9** | 80.95 |
| **33** | 23.50 | 11.02 | -53% | 120.5 | 1.32 | -99% | 3+ | 75.9 | 2+ | **60.0** | 57.69 |
| **34** | 27.47 | 29.32 | 7% | 308.7 |  |  |  |  |  |  |  |

**Supplementary Table 1.** **Evaluation of correlative autophagy markers.** Serum levels of HMGB1 and peripheral blood mononuclear cell LC3-II staining were evaluated pre and post treatment. These values in addition to the % change in response to treatment are reported for patients who had adequate amounts of serum available from the indicated time points. The resected pancreatic tumor specimen was immunohistochemically stained for several autophagy markers including Beclin 1, ATG 7 and CD68. Staining is reported as the % cancer cell staining and/or staining intensity. *PBMCs: peripheral blood mononuclear cells*
