# Supplementary material for: Encouraging long‐term survival following autophagy inhibition using neoadjuvant hydroxychloroquine and gemcitabine for high‐risk patients with resectable pancreatic carcinoma
Source: Cancer Med. 2021 Sep 24;10(20):7233–41. doi: 10.1002/cam4.4211 (PMC8525088; doi:10.1002/cam4.4211)
Supplement: Supplementary file 2 — Data S1 [file CAM4-10-7233-s001.docx]

**Supplemental Methods**

**Correlative Studies**

*Evaluation of Circulating Autophagy Markers.* Whole blood was collected for correlative studies before initiating and immediately following completion of therapy. Serum levels of HMGB1were measured using an ELISA from IBL International (Hamburg, Germany). HMGB1 is a nuclear transcription factor which is a damage associated molecular pattern molecule (DAMP). It is released by inflammatory and necrotic cells and acts as a marker of cell damage in the treatment groups [1-3]. Peripheral blood mononuclear cells (PBMCs) were isolated pre- and post-treatment using ficoll-hypaque density gradient centrifugation and stored in 2% paraformaldehyde.  Cytospin of PBMCs was performed and cells were stained for LC3-II using rabbit mAb to LC3-I/II (Novus, #100-2220, 5 ug/ml) in the presence of Triton X-100 at 0.1% at 4C. The slides were washed with 0.5% BSA, followed by a 1-hour incubation with a Cy3 secondary antibody (goat anti-rabbit, 1:1000, Jackson ImmunoResearch Laboratories) and Hoechst dye (1 mg/100 ml bisbenzimide) was applied for 30 s. The slides were rinsed with PBS and cover slipped with gelvatol, a water-soluble mounting media. Slides were then imaged with an Olympus Fluoview 1000 confocal scanning microscope (Olympus, Melville, NY). Imaging conditions were maintained at identical settings within each antibody-labeling experiment with original gating performed using the negative control. Quantification was performed using Metamorph (Molecular Devices, Sunnyvale, CA) to determine the mean fluorescent intensity (MFI) of punctate LC3-II adjusted for Hoechst dye MFI. The area of LC3 puncta per nucleus was averaged from at least 3 different fields.  Pre- and post-treatment LC3 staining was compared and correlated with clinical outcomes.  A LC3-II response was classified as having a change in LC3 from pre to post-treatment that was greater than 50%.

*Evaluation of Autophagy Markers in Pancreatic Tumors.*

Immunohistochemical staining for several autophagy markers was performed from resected pancreatic tumors using the following protocol. Briefly, paraffin embedded, formalin fixed pancreatic tumors were cut at 8µm sections. Slides were then heated at 56° C for 30 minutes, allowed to cool to room temperature, and then deparaffinized in xylene for 15 minutes twice. Slides were rehydrated in an ethanol gradient of 95% ethanol, 70% ethanol and water. Heat induced epitope retrieval or proteinase K treatment was used as appropriate for the individual antigen. Immunohistochemistry was performed on a Dako Autostainer with an Envision+HRP kit (Dako, catalog K4010) and a species matched secondary. Detection was completed with diaminobenzidine (Dako) and counter staining in Mayer’s Hematoxylin (Dako). Antibodies were obtained from the following manufacturers and staining was performed under the listed conditions: rabbit anti-beclin 1 (Sigma, #PRS3613, 1:2000, 30 min), rabbit anti-ATG 7 (Sigma, #HPA007639, 1:50, 30 min), mouse mAb anti-CD68 (Thermo, #MS-1808S1, 1:50, 30 min). A cover slip was applied to the slides with aqueous mounting medium (Faramount, Dako). Under 20x magnification, at least 3 different fields of carcinoma glandular structures were observed. The number of positive carcinoma cells divided by the total number of carcinoma cells examined was reported as the % cancer cell staining. The average intensity of staining was scored 0 to 4 based on the degree of staining in relation to other samples within in the group and to positive controls.

|  | **Correlative Studies - Serum** | | | **Correlative Studies - PBMCs** | | | **Correlative Studies-Resected Pancreas** | | | | |
| --- | --- | --- | --- | --- | --- | --- | --- | --- | --- | --- | --- |
|  | **HMGB1 ELISA ng/mL** | | | **LC3-II Cytospin Staining** | | | **Beclin 1** | | **ATG 7** | | **CD68** |
| **Pt ID** | **Pre Tx** | **Post Tx** | **% Change** | **Pre Tx** | **Post Tx** | **% Change** | **Staining intensity (0-4)** | **% Cancer cell staining** | **Staining intensity (0-4)** | **% Cancer cell staining** | **% Cancer cell staining** |
| **2** |  |  |  | 0.16 | 100.11 | 62469% | 2+ | 73.1 | 1+ | **58.3** | 32.26 |
| **3** |  |  |  | 52.89 | 565.37 | 969% | 2+ | 61.4 | 2+ | **60.7** | 50.00 |
| **4** |  |  |  | 339.65 | 410.13 | 21% | 0 - 1+ | 11.9 | <1+ | **21.2** | 63.49 |
| **5** |  |  |  | 261.08 | 395.45 | 51% |  |  |  |  |  |
| **7** | 2.91 | 4.96 | 70% | 921.51 | 3725.61 | 304% |  |  |  |  |  |
| **8** | 2.71 | 0.10 | -96% | 1001.04 | 278.65 | -72% | 0 - 1+ | 52.9 | 1+ | **52.2** | 19.23 |
| **9** | 6.01 | 13.25 | 120% | 1 | 353.29 | 35229% | 1+ | 25.0 | 1+ | **28.6** | 61.76 |
| **10** | 17.14 | 11.80 | -31% | 1681.6 |  |  | 2+ - 3+ | 82.5 | 2+ | **83.3** | 88.89 |
| **11** | 32.86 | 47.88 | 46% | 959.05 | 510.08 | -47% | 1+ - 2+ | 29.7 | 1+ | **75.0** | 75.76 |
| **12** | 6.70 | 4.74 | -29% | 169.83 | 472.88 | 178% | 2+ | 60.8 | 1+ | **48.3** | 43.33 |
| **13** | 10.75 | 0.57 | -95% |  |  |  | 1+ | 40.9 | 1+ - 2+ | **109.1** | 36.67 |
| **14** | 17.03 | 12.50 | -27% |  |  |  | 1+ | 49.2 | <1+ | **14.8** | 21.05 |
| **15** | 7.64 | 20.44 | 168% | 449.88 | 1437.86 | 220% | 0 - 1+ | 8.3 | 1+ - 2+ | **78.1** | 69.23 |
| **16** | 19.11 | 4.20 | -78% | 13.37 | 576.24 | 4210% |  |  |  |  |  |
| **17** |  |  |  | 542.8 |  |  | 2+ | 71.4 | 2+ | **83.3** | 33.33 |
| **18** | 5.05 | 6.03 | 19% | 131.02 | 91.38 | -30% | 1+ | 42.4 | 2+ | **66.7** | 96.77 |
| **19** |  |  |  | 169.9 |  |  | 1+ | 19.0 | 1+ | **70.8** | 66.67 |
| **20** |  |  |  | 304.0 |  |  | 2+ - 3+ | 47.4 | 0 - 1+ | **29.6** | 30.77 |
| **21** | 10.69 | 11.24 | 5% | 1604.73 | 23.78 | -99% | 1+ - 2+ | 43.2 | 2+ | **15.8** | 80.56 |
| **22** | 5.17 | 11.58 | 124% | 140.7 |  |  | 1+ - 3+ | 23.1 | 2+ | **91.9** | 59.26 |
| **23** |  |  |  | 182.9 |  |  | 2+ - 3+ | 69.4 | 2+ | **88.9** | 50.00 |
| **24** |  |  |  | 299.6 |  |  | 2+ | 40.0 | 3+ | **88.0** | 70.37 |
| **25** |  |  |  | 29.1 |  |  | 1+ - 2+ | 39.0 | 1+ | **53.8** | 34.62 |
| **27** |  |  |  |  |  |  |  |  |  |  |  |
| **29** | 11.31 | 7.62 | -33% | 4.57 | 130.92 | 2765% | 2+ | 27.3 | 0 - 1+ | **22.2** | 87.50 |
| **30** |  |  |  |  | 57.7 |  | 1+ - 3+ | 52.9 | 2+ | **92.9** | 25.00 |
| **31** | 19.26 | 11.15 | -42% | 89.5 | 3.92 | -96% | 0-3+ | 75.0 | 1+ | **90.9** | 80.95 |
| **33** | 23.50 | 11.02 | -53% | 120.5 | 1.32 | -99% | 3+ | 75.9 | 2+ | **60.0** | 57.69 |
| **34** | 27.47 | 29.32 | 7% | 308.7 |  |  |  |  |  |  |  |

**Supplementary Table 1.** **Evaluation of correlative autophagy markers.** Serum levels of HMGB1 and peripheral blood mononuclear cell LC3-II staining were evaluated pre and post treatment. These values in addition to the % change in response to treatment are reported for patients who had adequate amounts of serum available from the indicated time points. The resected pancreatic tumor specimen was immunohistochemically stained for several autophagy markers including Beclin 1, ATG 7 and CD68. Staining is reported as the % cancer cell staining and/or staining intensity. *PBMCs: peripheral blood mononuclear cells*

**REFERENCES**

1. Soloff AC, Jones KE, Powers AA, et al. HMGB1 Promotes Myeloid Egress and Limits Lymphatic Clearance of Malignant Pleural Effusions. *Front Immunol*. 2020:11:2027.
2. Mendonça Gorgulho C, Murthy P, et al. Different measures of HMGB1 location in cancer immunology. *Methods Enzymol*. 2019;629:195-217.
3. Gorgulho CM, Romagnoli GG, Bharthi R, et al. Johnny on the Spot-Chronic Inflammation Is Driven by HMGB1. Front Immunol. 2019;10:1561
